# Supplementary material for: CTLESS: A Scatter-Window Projection and Deep Learning-Based Transmission-Less Attenuation Compensation Method for Myocardial Perfusion SPECT
Source: IEEE Trans Med Imaging. Author manuscript; Available in PMC 2026 Jan 31. (PMC12860543; doi:10.1109/TMI.2024.3496870)
Supplement: Supplementary material [file NIHMS2066799-supplement-Supplementary_material.docx]

Supplementary Materials

CTLESS: A scatter-window projection and deep learning-based transmission-less attenuation compensation method for myocardial perfusion SPECT

Zitong Yu, *Student Member, IEEE*, Md Ashequr Rahman, *Student Member, IEEE*, Craig K. Abbey, Richard Laforest, Barry A. Siegel, Nancy A. Obuchowski, and Abhinav K. Jha, *Senior Member, IEEE*

## Synthetic defects-insertion procedure

We followed the same procedure in the study conducted by Narayanan et al. (Narayanan et al., *IEEE Trans. Nucl. Sci.*, 2001). We first segmented the left ventricle (LV) from the cardiac short-axis images using the Segment software. We located a 2-D cone region, of which the vertex is at the centroid of the LV wall, with specific extents and locations. In the slice contains the LV centroid, the LV wall lies in the 2-D cone region was considered as the defect mask. In the adjacent slices, the same cone region was used to create a 3-D defect. A 42 mm defect in the long-axis direction was considered. For each patient, we calculated the mean uptake in the LV region from the reconstructed image. We then assigned this uptake value in the defect masks with different severity levels. For example, if the mean uptake in the LV region of a patient was $\lambda_{LV}$, we assigned $\lambda_{LV}$×50% to the defect mask to create a 50% severity defect. Next, we reoriented the defect masks back to transverse slides and subtracted them from the reconstructed images. Thus, the reconstructed image with inserted defect was exactly similar to the reconstructed image without inserted defect, expect to those voxels corresponding to the defect region. We forward projected the reconstructed images both with and without the inserted defect, using the SIMIND Monte-Carlo (MC) simulation software, yielding the intermediate defect-present and data-absent projection data in the photopeak window, respectively. The MC-based forward projection operation simulated major relevant image-degrading effects in SPECT including attenuation, scatter, and collimator-detector response. Denote the intermediate defect-present and defect-absent projections by ${\hat{\mathbf{g}}}_{present}$ and ${\hat{\mathbf{g}}}_{absent}$, respectively. The intermediate projections were used to calculate the scaling-factor vector, denoted by $\mathbf{s}$, as shown below:

$$\begin{matrix} \mathbf{s}={\hat{\mathbf{g}}}_{present}⊘{\hat{\mathbf{g}}}_{absent}, \end{matrix} \left( 1 \right)$$

where $⊘$ is the Hadamard division. We then scaled the defect-absent photopeak-window projection data by $\mathbf{s}$, yielding the final defect-present projections.

## LV wall segmentation failure

N = 26 studies were excluded due to LV wall segmentation failure in the Segment software during the synthetic defect insertion. Representative examples are shown in Fig. S-1. The red circles indicate the endocardial border of LV wall segmented by Segment, and the green circles indicate the epicardial border of LV wall. The white crosses indicate the center of the actual LV wall. In those examples, the endocardial and epicardial borders of LV wall were not delineated correctly.


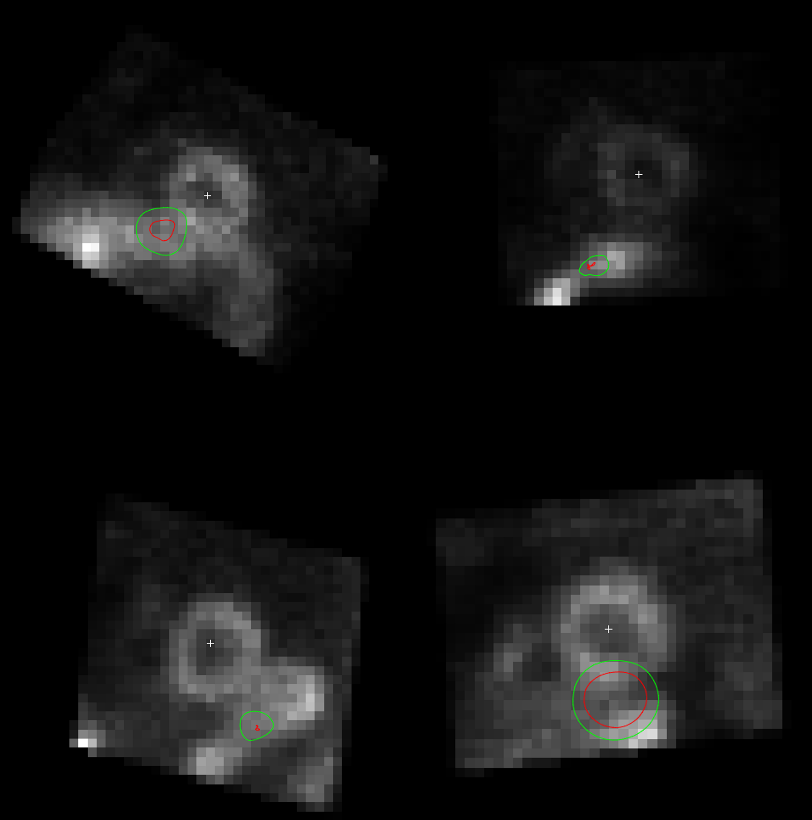


Fig. S-1. Representative examples of LV wall segmentation failure in the Segmentation software

## Detailed description of the McEUN

The McEUN consisted of two components, including an encoder and a assemble of six decoders. We provide detailed structures of the encoder and one decoder in the assemble as follows.

*Table S I. The architecture of the encoder.*

| **Index** | **Layer type** | **Input shape/layer** | **Kernel size** | **Stride size** | **Number of filters** | **Output shape** |
| --- | --- | --- | --- | --- | --- | --- |
| 1 | Input Layer | (64,64,64,2) | None | None | None | (64,64,64,2) |
| 2 | Conv 3D | (64,64,64,2) | (3,3,3) | (1,1,1) | 16 | (64,64,64,16) |
| 3 | Leaky ReLU | (64,64,64,16) | None | None | None | (64,64,64,16) |
| 4 | Dropout | (64,64,64,16) | None | None | None | (64,64,64,16) |
| 5 | Conv 3D | (64,64,64,16) | (3,3,3) | (2,2,2) | 32 | (32,32,32,32) |
| 6 | Leaky ReLU | (32,32,32,32) | None | None | None | (32,32,32,32) |
| 7 | Dropout | (32,32,32,32) | None | None | None | (32,32,32,32) |
| 8 | Conv 3D | (32,32,32,32) | (3,3,3) | (1,1,1) | 32 | (32,32,32,32) |
| 9 | Leaky ReLU | (32,32,32,32) | None | None | None | (32,32,32,32) |
| 10 | Dropout | (32,32,32,32) | None | None | None | (32,32,32,32) |
| 11 | Conv 3D | (32,32,32,32) | (3,3,3) | (2,2,2) | 64 | (16,16,16,64) |
| 12 | Leaky ReLU | (16,16,16,64) | None | None | None | (16,16,16,64) |
| 13 | Dropout | (16,16,16,64) | None | None | None | (16,16,16,64) |
| 14 | Conv 3D | (16,16,16,64) | (3,3,3) | (1,1,1) | 64 | (16,16,16,64) |
| 15 | Leaky ReLU | (16,16,16,64) | None | None | None | (16,16,16,64) |
| 16 | Dropout | (16,16,16,64) | None | None | None | (16,16,16,64) |

*Table S II. The architecture of one decoder in the assemble.*

| **Index** | **Layer type** | **Input shape/layer** | **Kernel size** | **Stride size** | **Number of filters** | **Output shape** |
| --- | --- | --- | --- | --- | --- | --- |
| 1 | TransConv 3D | (16,16,16,64) | (3,3,3) | (2,2,2) | 64 | (32,32,32,64) |
| 2 | Leaky ReLU | (32,32,32,64) | None | None | None | (32,32,32,64) |
| 3 | Dropout | (32,32,32,64) | None | None | None | (32,32,32,64) |
| 4 | Conv 3D | (32,32,32,64) | (3,3,3) | (1,1,1) | 64 | (32,32,32,64) |
| 5 | Leaky ReLU | (32,32,32,64) | None | None | None | (32,32,32,64) |
| 6 | Dropout | (32,32,32,64) | None | None | None | (32,32,32,64) |
| 7 | TransConv 3D | (32,32,32,64) | (3,3,3) | (2,2,2) | 32 | (64,64,64,32) |
| 8 | Leaky ReLU | (64,64,64,32) | None | None | None | (64,64,64,32) |
| 9 | Dropout | (64,64,64,32) | None | None | None | (64,64,64,32) |
| 10 | Conv 3D | (64,64,64,32) | (3,3,3) | (1,1,1) | 32 | (64,64,64,32) |
| 11 | Leaky ReLU | (64,64,64,32) | None | None | None | (64,64,64,32) |
| 12 | Dropout | (64,64,64,32) | None | None | None | (64,64,64,32) |
| 13 | TransConv 3D | (64,64,64,32) | (3,3,3) | (2,2,1) | 16 | (128,128,64,16) |
| 14 | Leaky ReLU | (128,128,64,16) | None | None | None | (128,128,64,16) |
| 15 | Dropout | (128,128,64,16) | None | None | None | (128,128,64,16) |
| 16 | Conv 3D | (128,128,64,16) | (3,3,3) | (1,1,1) | 16 | (128,128,64,16) |
| 17 | Leaky ReLU | (128,128,64,16) | None | None | None | (128,128,64,16) |
| 18 | Dropout | (128,128,64,16) | None | None | None | (128,128,64,16) |
| 19 | Conv 3D | (128,128,64,16) | (3,3,3) | (1,1,1) | 1 | (128,128,64) |
| 20 | Leaky ReLU | (128,128,64) | None | None | None | (128,128,64) |
| 21 | Dropout | (128,128,64) | None | None | None | (128,128,64) |

## The training and validation loss curves

The training and validation loss curves during the five-fold cross validation for the CTLESS method are shown in Fig. S-2.


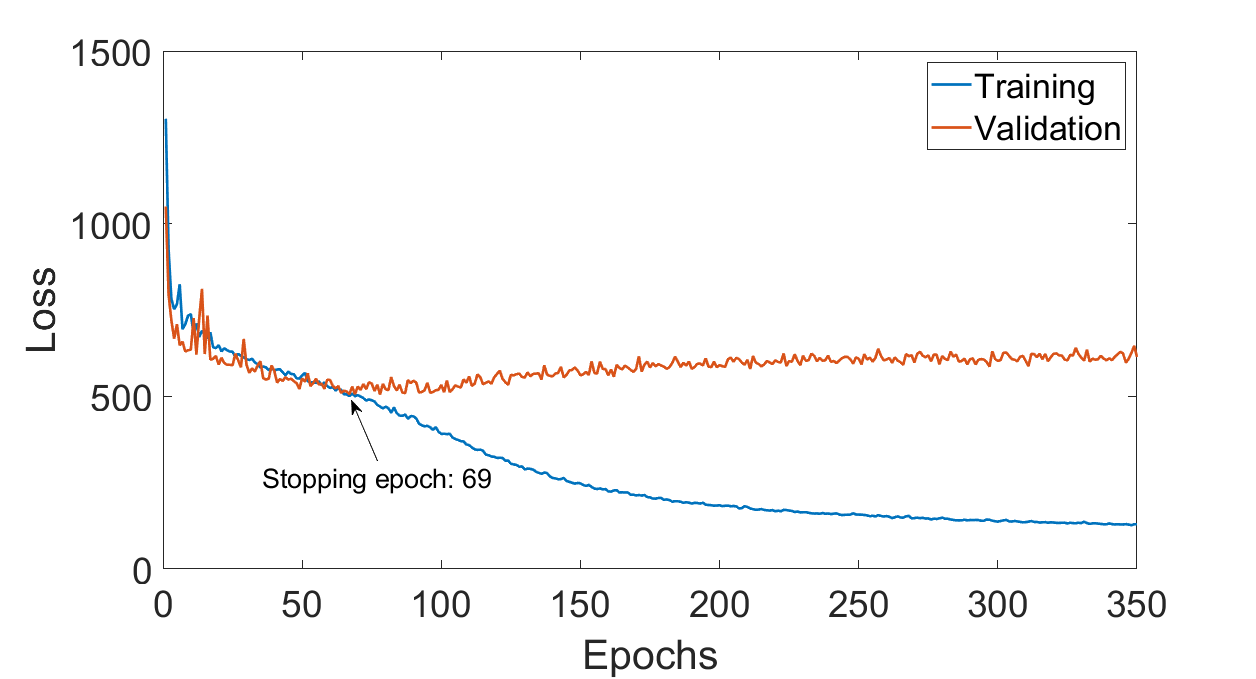


Fig. S-2. The training and validation loss curves during the five-fold cross validation.

## Optimized weight parameters in the loss function

We optimized the weight parameters in Eq. (7) via five-fold cross validation. The optimized weight parameters are listed in Table S III below.

*Table S III. Weight parameters*

| **Region** | **Weight** |
| --- | --- |
| Skin and subcutaneous adipose | 0.03 |
| Muscles and organs | 0.16 |
| Lungs | 0.32 |
| Bones | 0.32 |
| CT holder | 0.16 |
| Background | 0.01 |

## Details of EST-AC method

The EST-AC approach takes reconstructed images in scatter and photopeak windows as input and yields estimated attenuation coefficients of each voxel in the attenuation maps. The network contains one encoder and one decoder and has a leaky-ReLU activation function in the final layer. The structures of encoder and decoder are same as those of McEUN. The initial estimates of attenuation maps and the photopeak-energy window reconstruction without AC were normalized from [0, maximum pixel value in the image] to [0, 100], and then input to the network. The kernel weights of the McEUN were initialized using the Glorot normal initializer. Biases were initialized to a constant of 0.03. The McEUN was trained using Adam optimizer. The network was trained to minimize mean-square-error between estimated attenuation map and the CT-derived attenuation map via five-fold cross validation.

## Absolute p values in comparisons between AC methods

The absolute p-values in comparisons between AC methods are shown in Table S IV.

*Table S IV. Absolute p values in comparisons between AC methods*

| **Method 1** | **Method 2** | **Corresponding figure in the main manuscript** | **Null hypothesis** | **p value** |
| --- | --- | --- | --- | --- |
| CTAC | NAC | Fig. 6b | CTAC and NAC have the same AUC value. | $1.2\times{10}^{-8}$ |
| CTLESS | NAC | Fig. 6b | CTLESS and NAC have the same AUC value. | $6.5\times{10}^{-6}$ |
| CTLESS | CTAC | Fig. 7 | CTLESS is inferior to CTAC within the margin. | $1.2\times{10}^{-9}$ |
| NAC | CTAC | Fig. 7 | NAC is inferior to CTAC within the margin. | $0.9947$ |
| CTAC | NAC | Fig. 8: Female | CTAC and NAC have the same AUC value in female patients. | $<{10}^{-10}$ |
| CTLESS | NAC | Fig. 8: Female | CTLESS and NAC have the same AUC value in female patients. | $<{10}^{-10}$ |
| CTAC | NAC | Fig. 8: Male | CTAC and NAC have the same AUC value in male patients. | $1.4\times{10}^{-9}$ |
| CTLESS | NAC | Fig. 8: Male | CTLESS and NAC have the same AUC value in male patients. | $1.0\times{10}^{-7}$ |
| CTAC | NAC | Fig. 9a | CTAC and NAC have the same AUC value. | $<{10}^{-10}$ |
| CTLESS  (trained on CZT) | NAC | Fig. 9a | CTLESS and NAC have the same AUC value. | $<{10}^{-10}$ |
| CTLESS  (trained on NaI) | NAC | Fig. 9a | CTLESS and NAC have the same AUC value. | $<{10}^{-10}$ |
| CTAC | NAC | Fig. 9b | CTAC and NAC have the same AUC value. | $<{10}^{-10}$ |
| CTLESS  (trained on CZT) | NAC | Fig. 9b | CTLESS and NAC have the same AUC value. | $<{10}^{-10}$ |
| CTLESS  (trained on NaI) | NAC | Fig. 9b | CTLESS and NAC have the same AUC value. | $6.6\times{10}^{-8}$ |
| CTLESS | NAC | Fig. 11: RMSE | CTLESS and NAC have the same RMSE. | $<{10}^{-10}$ |
| CTLESS | NAC | Fig. 11: SSIM | CTLESS and NAC have the same SSIM. | $<{10}^{-10}$ |
